# Detection of *Nocardia* by 16S Ribosomal RNA Gene PCR and Metagenomic Next-Generation Sequencing (mNGS)

**DOI:** 10.3389/fcimb.2021.768613

**Published:** 2022-01-07

**Authors:** Juanjuan Ding, Bing Ma, Xupeng Wei, Ying Li

**Affiliations:** ^1^ Department of Respiratory and Critical Care Medicine, Henan Provincial People’s Hospital, Zhengzhou, China; ^2^ Department of Clinical Laboratory, Henan Provincial People’s Hospital, Zhengzhou, China; ^3^ Department of Respiratory and Critical Care Medicine, Xuchang Central Hospital, Xuchang, China

**Keywords:** nocardiosis, 16S ribosomal RNA gene, polymerase chain reaction, next generation sequencing, species, molecular diagnosis

## Abstract

In this study, the aim was to investigate the discriminatory power of molecular diagnostics based on mNGS and traditional 16S ribosomal RNA PCR among *Nocardia* species. A total of fourteen clinical isolates from patients with positive *Nocardia* cultures and clinical evidence were included between January 2017 and June 2020 in HeNan Provincial People’s Hospital. DNA extraction and 16S rRNA PCR were performed on positive cultures, and pathogens were detected by mNGS in these same samples directly. Among the 14 *Nocardia* isolates, four species were identified, and *N. cyriacigeorgica* (8 cases) is the most common species. Twelve of the 14 *Nocardia* spp. isolates were identified by the two methods, while two strains of *N. cyriacigeorgica* were not identified by mNGS. All tested isolates showed susceptibility to trimethoprim-sulfamethoxazole (SXT), amikacin and linezolid. Apart from *Nocardia* species, other pathogens such as *Acinetobacter baumannii, Klebsiella pneumonia, Aspergillus, Enterococcus faecalis, Human herpesvirus*, etc., were detected from the same clinical samples by mNGS. However, these different pathogens were considered as colonization or contamination. We found that it is essential to accurately identify species for determining antibiotic sensitivity and, consequently, choosing antibiotic treatment. 16S rRNA PCR was useful for identification of nocardial infection among species, while this technique needs the clinicians to make the pre-considerations of nocardiosis. However, mNGS may be a putative tool for rapid and accurate detection and identification of *Nocardia*, beneficial for applications of antimicrobial drugs and timely adjustments of medication.

## Introduction

*Nocardia* is a genus of aerobic, Gram-positive, branching, filamentous, weakly acid-fast bacteria (Wang et al., 2015). The organisms are saprophytic and are found in water, soil, dust, and vegetable matter. Upon exposure to dust particles, *Nocardia* may become airborne, causing infection by inhalation (Chen et al., 2014). Individuals with poor immune system, for example, patients with lung disorders, diabetes, cancers, HIV/AIDS, chronic alcoholism, connective tissue disorders, organ transplantation, and those undergoing corticosteroid therapy are prone to infections with *Nocardia* spp. (Kandi, 2015). The major clinical symptoms of human nocardiosis include lymphadenitis, lymphangitis, encephalitis, pneumonia, and cutaneous tissue lesions (Condas et al., 2015). Despite its infrequency, pulmonary nocardiosis is clinically severe (Sadamatsu et al., 2017). Recently, nocardiosis has been described as isolated cases and case series in lungs and other sites of immunocompetent patients, representing 33%-56% of total cases. Of note, bronchiectasis, chronic obstructive pulmonary disease (COPD), pulmonary alveolar proteinosis, and asthma are predisposing factors among patients with chronic respiratory diseases (Sadamatsu et al., 2017).

Reports on the incidence of *Nocardia* infection worldwide are limited, which might be caused by difficulties in isolation and incubation of *Nocardia* as well as lack of systematic literatures on *Nocardia* infection in health system (Darazam et al., 2013). There are annually approximate 500-1000 cases of nocardiosis in the United States. 16S rRNA PCR is vital for the accurate identification of *Nocardia* in clinical microbiology laboratories, but this method requires *a priori* knowledge of microorganisms that are suspected to exist in clinical samples under investigation prior to detection (Boers et al., 2019). Unbiased metagenomic next generation sequencing (mNGS) can detect infectious especially the rare or new pathogens (Lu et al., 2020), and compared with traditional methods, it performs better in diagnosis, indicating its application in early diagnosis and management of patients.

However, understanding of nocardiosis is mostly derived from case reports in China, and case series are required for shedding light on nocardiosis. We conducted this study to identify *Nocardia* species using 16SrRNA and mNGS in a tertiary hospital in China. The major aim was to investigate the discriminatory power of molecular diagnostics based on mNGS and traditional 16S ribosomal RNA PCR among *Nocardia* species. A secondary objective was to investigate the species distribution, clinical manifestations, and microbiological characteristics in our hospital.

## Methods

### Study Design and Participants

This study was conducted at the HeNan Provincial People’s Hospital, a 3900-bed tertiary care teaching hospital in China and all culture-proven cases of nocardiosis from January 2017 to June 2020. Medical records were reviewed for clinical information, including demographics, laboratory and radiological findings, physical examination, symptoms at presentation, antibiotic treatment, and outcome. Isolation of *Nocardia* species from clinical samples including bronchoalveolar lavage, sputum, lung aspiration, and purulent pus as well as pertinent clinical symptoms and imaging findings were required for nocardiosis diagnosis. *Nocardia* species were identified by conventional phenotypic identification methods and Bruker MALDI-TOF MS systems (Bruker Daltonics, Billerica, MA, USA). After the microbial culture dish gave an alarm, parallel specimen were sent to commercial companies for mNGS. 16S rRNA PCR was carried out to identify the species for the culture results. In spite of *Nocardia* being isolated from samples, cases without clinical symptoms and chest radiographical findings were judged as colonization and excluded from analysis. Disseminated nocardiosis was defined as involvement of ≥2 non-contiguous organs or the central nervous system.

### 16S rRNA PCR and Sequencing

The bacteria isolation and culture protocol were conducted according to a previous study (Larruskain et al., 2011). The species further identification of each *Nocardia* strain was done by sequencing the 16S rRNA gene (1500 bp).Genomic DNA of *Nocardia* was extracted by using Ezup Column Bacteria Genomic DNA Purification Kit (Cat. No. SK8255, Sangon, China). PCR primers were the bacterial 16S rRNA universal primer pairs, 7F and 1540R(5’ CAGAGTTTGATCCTGGCT3’, 5’AGGAGGTGATCCAGCCGCA3’), 27F and 1492R(5’ AGTTTGATCTTGGCTCAG 3’, 5’GGTTACCTTGTTACGACTT 3’). The 16S rRNA gene was amplified by PCR under the thermal cycling conditions of 35 cycles at 94°C for 1 min for denaturation, 60°C for 1 min for primer annealing, and 72°C for 2 min for primer extension. A 3730XL automated DNA sequencer (Applied Biosystems, Foster City, CA, USA) with primers used in gene amplification was used for sequencing the PCR products. The resulting DNA sequences were analyzed by the BLAST program.

### Metagenomic Next Generation Sequencing and Data Analysis

A 300 μl sample of bronchoalveolar lavage fluid (BALF), sputum, tissue, purulent pus, etc. were collected in DNase/RNase tubes for identifying the potential pathogens. DNA libraries were constructed through transposase mediated methods, and PCR amplification followed (Vision Medicals, China). For measurement of the adapters and the sizes of fragments before sequencing, the quality of the DNA libraries was determined by an Qsep1 bio-fragment analyzer (BiOptic. Inc., La Canada Flintridge, CA). Qualified DNA libraries were pooled together and sequenced on Nextseq 550 Dx sequencing platform (illumina, San Diego, CA). After removal of low-quality, and short (length < 40bp) reads, Burrows-Wheeler Alignment was used for computational subtraction of human host sequences mapped to the human reference genome (hg38 and YH sequences), and the high-quality sequencing data were generated. After removal of low-complexity reads, the remaining data were classified according to four (bacteria, parasites, fungi, viruses) Microbial Genome Databases. The classification reference databases were downloaded and optimized from public databases including NCBI, EBI or Genbank. At last, the multi-parameters of species in Microbial Genome Databases were calculated and exported. Results were interpreted by professionals with microbiology and clinical background.

### Antimicrobial Susceptibility Tests

The *in vitro* antimicrobial susceptibility of all strains was detected using broth microdilution method, as recommended by the CLSI for antimicrobial susceptibility testing of *Nocardia*. Antimicrobials (amikacin, trimethoprim-sulfamethoxazole, linezolid, imipenem, minocycline, cefotaxime, ciprofloxacin, tobramycin) were used for susceptibility testing. Resistance to two or more of the most commonly used drugs (amikacin, ceftriaxone, TMP-SMZ and imipenem) refers to multidrug resistance (Betrán et al., 2016).

## Results

### Demographic and Clinical Features

In the present study, fourteen patients with positive *Nocardia* cultures and clinical evidence were included. The clinical features of the patients and *Nocardia* strains isolated from samples are shown in **Table 1**. In 12 cases of these patients, only the lung was infected. One case had pulmonary and neurological involvement. The other patient had skin involvement. Male patients accounted for nine cases, and the median age was 56 years (range, 23-75) in all fourteen patients. Chronic lung disease (6 cases), including bronchiectasis (4 cases), COPD (1 case) and interstitial pneumonia (1 case), were most common underlying diseases. Other 7 patients had underlying disease with poor immune system, including solid tumor (3 cases), chronic renal disease (2 cases), diabetes mellitus (1 case) and hematological malignancy (1 case). Only one patient with skin nocardiosis had no underlying disease. Three patients died of the severity of their disease. The most common presenting manifestations were fever (8 cases), cough (7 cases) and sputum production (5 cases), followed by dyspnea (4 cases), hemoptysis (1 cases), chest pain (1 case), cutaneous abscess (1 case). Neurological signs were observed in one patient who has disseminated nocardiosis. All 14 patients underwent chest computed tomography (CT). Bronchiectasis (n=4) and infiltration (n=4) were the most common presentation of chest CT findings, followed by consolidation (n=3), masses (n=3), cavity (n=3), nodules (n=2) and pleural effusion (n=1).

**Table 1:** Clinical features of the patients and *Nocardia* strains isolated from samples.

No.	Age	Gender	Underlying disease	Symptoms	Imaging	Specimen	Species by 16S rRNA	Identify	Referrence Assesion	Species by mNGS	Antibiotic treatment	Outcome
1	75	male	Lung cancer	cough	mass	lung aspiration	*N.cyriacigeorgica*	100%	LR215973.1	*N.cyriacigeorgica*	SXT	Recovery
2	53	male	Membranous nephropathy	fever, dyspnea	nodule;infiltration	suputum	*N.cyriacigeorgica*	100%	LR215973.1	*N.cyriacigeorgica*	SXT+IPM	Recovery
3	49	female	Bronchiectasis	fever, cough, sputum production	bronchiectasis, infiltration	BAL	*N. facinica*	100%	MN100049.1	*N. facinica*	SXT	Recovery
4	23	female	Bronchiectasis	cough, sputum production	bronchiectasis, infiltration	BAL	*N. facinica*	100%	MN100049.1	*N. facinica*	SXT	Recovery
5	42	female	Diabetes mellitus	fever, cough, sputum production	consolidation, pleural effusion	BAL	*N.otitidiscaviarum*	100%	KX500116.1	*N.otitidiscaviarum*	SXT	Death
6	68	male	Nephrotic syndrome	fever, hemoptysis, chest pain.neurological signs	cavity	BAL	*N.cyriacigeorgica*	100%	LR215973.1	Unidentified	SXT+IPM+ LZD	Death
7	70	male	COPD	dyspnea	cavity, mass	suputum	*N.brasiliensis*	99.93%	CP022088.2	*N.brasiliensis*	SXT+ LVX	Recovery
8	50	male	Bronchiectasis	fever, cough, suputum production,	bronchiectasis, infiltration	BAL	*N.cyriacigeorgica*	100%	LR215973.1	Unidentified	SXT	Recovery
9	78	male	None	cutaneous abscess	none	purulent pus	*N.cyriacigeorgica*	100%	LR215973.1	*N.cyriacigeorgica*	SXT	Recovery
10	56	female	Interstitial pneumonia	dyspnea,	consolidation	lung aspiration	*N.cyriacigeorgica*	100%	LR215973.1	*N.cyriacigeorgica*	SXT	Recovery
11	68	female	Lung cancer	fever, dyspnea	mass,	suputum	*N.cyriacigeorgica*	100%	LR215973.1	*N.cyriacigeorgica*	SXT	Recovery
12	28	male	Hematological malignancy	fever, cough	consolidation	BAL	*N. facinica*	100%	MN100049.1	*N. facinica*	SXT+IPM+ LZD	Death
13	63	male	Esophageal cancer	fever	nodules,cavity	BAL	*N.cyriacigeorgica*	100%	LR215973.1	*N.cyriacigeorgica*	SXT+ LVX	Recovery
14	57	male	Bronchiectasis	cough, sputum production	bronchiectasis,	BAL	*N. facinica*	100%	MN100049.1	*N. facinica*	SXT	Recovery

BAL, brochoalveolar lavage; COPD, chronic obstructive pulmonary disease; SXT, trimethoprim-sulfamethoxazole; IPM, imipenem; LZD, linezolid; LVX, levofloxacin.

### Distribution of *Nocardia* Species

Of the 14 *Nocardia* isolates, 12 isolates were identified by the two methods, while two isolates of *N. cyriacigeorgica* were not identified by mNGS. The isolated *Nocardia* species include *N. cyriacigeorgica* (8 cases), *N. farcinic*a (4 cases), *N. otitidiscaviarum* (1 case) and *N. brasiliensis* (1 case), determined by its 99.93% or 100% sequence similarity to the reference sequence in GenBank (**Table 1**).

### Antimicrobial Susceptibility

Tests of antimicrobial susceptibility were performed on these isolates, and the results are provided in **Table 2**. All tested isolates were susceptible to amikacin, trimethoprim-sulfamethoxazole, linezolid. Susceptible isolates were seen for imipenem (10/14), minocycline (8/14), cefotaxime (4/14) and ciprofloxacin (6/14). Multidrug-resistance was found in four isolates including one isolate of *N. cyriacigeorgica* (1/8), one isolate of *N. farcinica* (1/4), one isolate of *N. otitidiscaviarum* (1/1) and one isolate of *N. brasiliensis* (1/1). All these four multidrug-resistant isolates were resistant to imipenem, cefotaxime, ciprofloxacin,

**Table 2:** Antimicrobial susceptibility of *Nocardia* species was determined using the standard broth microdilution method.

Antimicrobials	No. of susceptible isolates				
	*N. cyriacigeorgica* (n=8)	*N. facinica* (n=4)	*N.otitidiscaviarum* (n=1)	*N.brasiliensis* (n=1)	Total (n=14)
Amikacin	8	4	1	1	14
TMP-SMZ	8	4	1	1	14
linezolid	8	4	1	1	14
Imipenem	7	3	0	0	10
Minocycline	3	3	1	1	8
cefotaxime	3	1	0	0	4
Ciprofloxacin	3	3	0	0	6
Tobramycin	6	0	1	1	8

### Other Pathogens Detected by mNGS

Other than *Nocardia* species, different pathogens such as *Acinetobacter baumannii*, *Klebsiella pneumonia, Aspergillus, Enterococcus faecalis, Human herpesvirus*, etc., were detected from the same clinical samples by mNGS. Combined with clinical manifestations, conventional culture method and (1, 3) -β-D-glucan and galactomannan (GM) test, these different pathogens were considered as colonization or contamination.

## Discussion

Although being considered as an opportunistic infection, nocardiosis also infects immunocompetent hosts. In this study, the most common underlying disease is bronchiectasis, which is consistent with the previous report from China. A study by Yang et al. that analyzed 13 patients infected by nocardiosis showed that twelve cases were diagnosed with pulmonary nocardiosis and bronchiectasis in 6 patients (Yang et al., 2017). Results from other reports are different, showing that COPD is the third most common risk factor, second only to chronic steroid therapy and solid organ transplantation (Kancherla et al., 2019). Nevertheless, bronchiectasis and COPD were deemed as risk factors for pulmonary nocardiosis (Kancherla et al., 2019). Pulmonary structural abnormalities and bacterial colonization on the bronchus facilitate the presence of *Nocardia* (Tomás et al., 2007). Patients with bronchiectasis were increasingly diagnosed with pulmonary nocardiosis. The reasons for the apparent increase are not entirely clear. The increased incidence of nocardiosis with bronchiectasis can be caused by environmental exposures, microbiologic surveillance, and other factors (Woodworth et al., 2017).

Clinical manifestations of nocardiosis lack specificity (Mootsikapun et al., 2005). Our findings indicated that the most common clinical manifestations were fever and cough. In this study, bronchiectasis and infiltration were the most common CT manifestations, followed by consolidation, masses, cavity, nodules and pleural effusion. Different from our study, most patients were observed with cavitation coupled with nodules, masses and consolidations in other reports (Chen et al., 2014). Mehrian et al. found that multiple pulmonary nodules, consolidation, and cavity are the main CT presentations of pulmonary nocardiosis in either immunocompetent patients or those with immune deficit, but these characteristics are not specific (Mehrian et al., 2015). The higher prevalence of bronchiectasis in our study in comparison to other reports is probably the main reason for the difference of computed tomography features.

In consistent with other studies, the *Nocardia* species showed good sensitivity to SXT, linezolid, and amikacin in the present study. Additionally, these isolates showed varying susceptibilities to different antibiotics. Different *Nocardia* species may have different susceptibility profiles, and this information is crucial for providing adequate antimicrobial therapy as well as investigating the epidemiology of *Nocardia* infections (Tamakoshi et al., 2018). When *Nocardia* is isolated from clinical specimens, the species should be identified. The traditional culture and biochemical methods are very time-consuming, resulting in a delayed diagnosis of nocardiosis. However, early application of suitable antibiotherapy, which depends on early detection of the nocardial infection, is vital for decreasing the high death rates of patients with nocardiosis (Couble et al., 2005). 16S rRNA PCR and sequencing offer accurate, inexpensive, rapid, and reliable identification of nocardial infection among species (Rahdar et al., 2021). At present, 16S rRNA PCR and sequencing are used for limited number of pathogens at a time or successful culture of microorganisms from clinical samples was required. In our study, 16S ribosomal RNA gene sequencing was useful for identification and detection of species of *Nocardia.* However, two isolates of *N. cyriacigeorgica* were not identified by mNGS. The possible reason is that mNGS currently has some limitations, such as human background and lack of unified standards for detailed experimental steps. However, since mNGS possesses more sensitivity than 16S rRNA sequencing analysis in diagnosing unspecified pathogens, especially rare pathogens, mNGS was adopted for pathogenic diagnosis of diseases. In our study, mNGS detected other pathogens apart from *Nocardia* species in the same samples, nevertheless, these cases were not considered as mixed infection. The results of mNGS still should be analyzed by clinicians in combination with the clinical features and conventional methods (such as traditional culture, G test, GM test, Aspergillus IgG antibody test, Cryptococcus serum antigen assay, etc.). Almost all pathogens in the samples can be detected by mNGS, while 16S rRNA PCR and sequencing were only applied for detection of a specific pathogen with the matched primers, and under such condition, rare pathogens may be missed, or primers containing mismatches for the tested pathogens may be mistakenly used, which leads to reduction in sensitivity and failure in detection (Gu et al., 2019). Thus, mNGS combined with targeted sequencing may be significantly useful for nonsterile samples, such as those from stool, polymicrobial abscesses, or bronchoalveolar lavage (Gu et al., 2019).

In conclusion, our study analyzed imaging findings, clinical features, drug susceptibility pattern, treatment and outcomes of nocardiosis-infected patients. These results will be promising for early diagnosis and management of patients. We found that 16S rRNA PCR was useful for identification of nocardial infection among species, while this technique needs the pre-considerations of nocardiosis by clinicians. However, mNGS may be advantageous for rapid and accurate detection and identification of *Nocardia*, which promotes applications of appropriate antimicrobial drugs and timely medication adjustments if necessary.

## Data Availability Statement

All data have been uploaded to China National GeneBank DataBase (http://db.cngb.org/cnsa), CNP0002551.

## Ethics Statement

The studies involving human participants were reviewed and approved by Ethics Review Committee of Henan Provincial People’s Hospital. The patients/participants provided their written informed consent to participate in this study.

## Author Contributions

JD designed the study, collected and analyzed the data, wrote, and edited the manuscript. BM collected the samples, participated in the next generation sequencing, and edited the manuscript. XW collected the samples and data. YL collected and analyzed the data, wrote, and edited the manuscript.

## Funding

This work was supported by Project from Science and Technology of Henan Province (132300410061).

## Conflict of Interest

The authors declare that the research was conducted in the absence of any commercial or financial relationships that could be construed as a potential conflict of interest.

## Publisher’s Note

All claims expressed in this article are solely those of the authors and do not necessarily represent those of their affiliated organizations, or those of the publisher, the editors and the reviewers. Any product that may be evaluated in this article, or claim that may be made by its manufacturer, is not guaranteed or endorsed by the publisher.
