# Supplementary material for: Detection of Nocardia by 16S Ribosomal RNA Gene PCR and Metagenomic Next-Generation Sequencing (mNGS)
Source: Front Cell Infect Microbiol. 2022 Jan 7;11:768613. doi: 10.3389/fcimb.2021.768613 (PMC8779735; doi:10.3389/fcimb.2021.768613)
Supplement: Supplementary file 1 [file Table_1.docx]

**No.1 *Nocardia cyriacigeorgica* 1465bp**

AACGCTGGCGGCGTGCTTAACACATGCAAGTCGAGCGGTAAGGCCCTTCGGGGTACACGAGCGGCGAACGGGTGAGTAACACGTGGGTGATCTGCCTCGCACTCTGGGATAAGCCTGGGAAACTGGGTCTAATACCGGATATGACCTTACATCGCATGGTGTTTGGTGGAAAGATTTATCGGTGCGAGATGGGCCCGCGGCCTATCAGCTTGTTGGTGGGGTAATGGCCTACCAAGGCGACGACGGGTAGCCGGCCTGAGAGGGCGACCGGCCACACTGGGACTGAGACACGGCCCAGACTCCTACGGGAGGCAGCAGTGGGGAATATTGCACAATGGGCGAAAGCCTGATGCAGCGACGCCGCGTGAGGGATGACGGCCTTCGGGTTGTAAACCTCTTTCGACAGGGACGAAGCGCAAGTGACGGTACCTGTAGAAGAAGCACCGGCCAACTACGTGCCAGCAGCCGCGGTAATACGTAGGGTGCGAGCGTTGTCCGGAATTACTGGGCGTAAAGAGCTTGTAGGCGGCTTGTCGCGTCGATCGTGAAAACTTGGGGCTCAACCCCAAGCTTGCGGTCGATACGGGCAGGCTTGAGTACTTCAGGGGAGACTGGAATTCCTGGTGTAGCGGTGAAATGCGCAGATATCAGGAGGAACACCGGTGGCGAAGGCGGGTCTCTGGGAAGTAACTGACGCTGAGAAGCGAAAGCGTGGGTAGCGAACAGGATTAGATACCCTGGTAGTCCACGCCGTAAACGGTGGGTACTAGGTGTGGGTTTCCTTCCACGGGATCCGTGCCGTAGCTAACGCATTAAGTACCCCGCCTGGGGAGTACGGCCGCAAGGCTAAAACTCAAAGGAATTGACGGGGGCCCGCACAAGCGGCGGAGCATGTGGATTAATTCGATGCAACGCGAAGAACCTTACCTGGGTTTGACATACACCGGAAACCTGCAGAGATGTAGGCCCCCTTGTGGTCGGTGTACAGGTGGTGCATGGCTGTCGTCAGCTCGTGTCGTGAGATGTTGGGTTAAGTCCCGCAACGAGCGCAACCCTTATCTTATGTTGCCAGCGCGTAATGGCGGGGACTCGTGAGAGACTGCCGGGGTCAACTCGGAGGAAGGTGGGGACGACGTCAAGTCATCATGCCCCTTATGTCCAGGGCTTCACACATGCTACAATGGCCGGTACAGAGGGCTGCGATACCGTGAGGTGGAGCGAATCCCTTAAAGCCGGTCTCAGTTCGGATCGGGGTCTGCAACTCGACCCCGTGAAGTTGGAGTCGCTAGTAATCGCAGATCAGCAACGCTGCGGTGAATACGTTCCCGGGCCTTGTACACACCGCCCGTCACGTCATGAAAGTCGGTAACACCCGAAGCCGGTGGCCTAACCCCTTGTGGGAGGGAGCCGTCGAAGGTGGGATCGGCGATTGGGACGAAGTCGTAACAAGGTAGCCGTACCGGAA

**No.2 *Nocardia cyriacigeorgica* 1449bp**

GCGTGCTTAACACATGCAAGTCGAGCGGTAAGGCCCTTCGGGGTACACGAGCGGCGAACGGGTGAGTAACACGTGGGTGATCTGCCTCGCACTCTGGGATAAGCCTGGGAAACTGGGTCTAATACCGGATATGACCTTACATCGCATGGTGTTTGGTGGAAAGATTTATCGGTGCGAGATGGGCCCGCGGCCTATCAGCTTGTTGGTGGGGTAATGGCCTACCAAGGCGACGACGGGTAGCCGGCCTGAGAGGGCGACCGGCCACACTGGGACTGAGACACGGCCCAGACTCCTACGGGAGGCAGCAGTGGGGAATATTGCACAATGGGCGAAAGCCTGATGCAGCGACGCCGCGTGAGGGATGACGGCCTTCGGGTTGTAAACCTCTTTCGACAGGGACGAAGCGCAAGTGACGGTACCTGTAGAAGAAGCACCGGCCAACTACGTGCCAGCAGCCGCGGTAATACGTAGGGTGCGAGCGTTGTCCGGAATTACTGGGCGTAAAGAGCTTGTAGGCGGCTTGTCGCGTCGATCGTGAAAACTTGGGGCTCAACCCCAAGCTTGCGGTCGATACGGGCAGGCTTGAGTACTTCAGGGGAGACTGGAATTCCTGGTGTAGCGGTGAAATGCGCAGATATCAGGAGGAACACCGGTGGCGAAGGCGGGTCTCTGGGAAGTAACTGACGCTGAGAAGCGAAAGCGTGGG-TAGCGAACAGGATTAGATACCCTGGTAGTCCACGCCGTAAACGGTGGGTACTAGGTGTGGGTTTCCTTCCACGGGATCCGTGCCGTAGCTAACGCATTAAGTACCCCGCCTGGGGAGTACGGCCGCAAGGCTAAAACTCAAAGGAATTGACGGGGGCCCGCACAAGCGGCGGAGCATGTGGATTAATTCGATGCAACGCGAAGAACCTTACCTGGGTTTGACATACACCGGAAACCTGCAGAGATGTAGGCCCCCTTGTGGTCGGTGTACAGGTGGTGCATGGCTGTCGTCAGCTCGTGTCGTGAGATGTTGGGTTAAGTCCCGCAACGAGCGCAACCCTTATCTTATGTTGCCAGCGCGTAATGGCGGGGACTCGTGAGAGACTGCCGGGGTCAACTCGGAGGAAGGTGGGGACGACGTCAAGTCATCATGCCCCTTATGTCCAGGGCTTCACACATGCTACAATGGCCGGTACAGAGGGCTGCGATACCGTGAGGTGGAGCGAATCCCTTAAAGCCGGTCTCAGTTCGGATCGGGGTCTGCAACTCGACCCCGTGAAGTTGGAGTCGCTAGTAATCGCAGATCAGCAACGCTGCGGTGAATACGTTCCCGGGCCTTGTACACACCGCCCGTCACGTCATGAAAGTCGGTAACACCCGAAGCCGGTGGCCTAACCCCTTGTGGGAGGGAGCCGTCGAAGGTGGGATCGGCGATTGGGACGAAGTCGTAACAAGGTAGCCGT

**No.3 *Nocardia farcinia* 1452bp**

CTGGCGGCGTGCTTAACACATGCAAGTCGAGCGGTAAGGCCCTTCGGGGTACACGAGCGGCGAACGGGTGAGTAACACGTGGGTGATCTGCCCTGTACTTCGGGATAAGCCTGGGAAACTGGGTCTAATACCGGATATGACCTTACATCGCATGGTGTTTGGTGGAAAGATTTATCGGTACAGGATGGGCCCGCGGCCTATCAGCTTGTTGGTGGGGTAATGGCCTACCAAGGCGACGACGGGTAGCCGGCCTGAGAGGGCGACCGGCCACACTGGGACTGAGACACGGCCCAGACTCCTACGGGAGGCAGCAGTGGGGAATATTGCACAATGGGCGAAAGCCTGATGCAGCGACGCCGCGTGAGGGATGACGGCCTTCGGGTTGTAAACCTCTTTCGACAGGGACGAAGCGCAAGTGACGGTACCTGTAGAAGAAGCACCGGCCAACTACGTGCCAGCAGCCGCGGTAATACGTAGGGTGCGAGCGTTGTCCGGAATTACTGGGCGTAAAGAGCTTGTAGGCGGTTTGTCGCGTCGTCCGTGAAAACTTGGGGCTCAACCCCAAGCTTGCGGGCGATACGGGCAGACTTGAGTACTGCAGGGGAGACTGGAATTCCTGGTGTAGCGGTGAAATGCGCAGATATCAGGAGGAACACCGGTGGCGAAGGCGGGTCTCTGGGCAGTAACTGACGCTGAGAAGCGAAAGCGTGGGTAGCGAACAGGATTAGATACCCTGGTAGTCCACGCCGTAAACGGTGGGCGCTAGGTGTGGGTTTCCTTCCACGGGATCCGTGCCGTAGCTAACGCATTAAGCGCCCCGCCTGGGGAGTACGGCCGCAAGGCTAAAACTCAAAGGAATTGACGGGGGCCCGCACAAGCGGCGGAGCATGTGGATTAATTCGATGCAACGCGAAGAACCTTACCTGGGTTTGACATACACCGGAAACCTGCAGAGATGTAGGCCCCCTTGTGGTCGGTGTACAGGTGGTGCATGGCTGTCGTCAGCTCGTGTCGTGAGATGTTGGGTTAAGTCCCGCAACGAGCGCAACCCTTGTCCTGTGTTGCCAGCGCGTTATGGCGGGGACTCGCAGGAGACTGCCGGGGTCAACTCGGAGGAAGGTGGGGACGACGTCAAGTCATCATGCCCCTTATGTCCAGGGCTTCACACATGCTACAATGGCCGGTACAGAGGGCTGCGATACCGTGAGGTGGAGCGAATCCCTTAAAGCCGGTCTCAGTTCGGATCGGGGTCTGCAACTCGACCCCGTGAAGTTGGAGTCGCTAGTAATCGCAGATCAGCAACGCTGCGGTGAATACGTTCCCGGGCCTTGTACACACCGCCCGTCACGTCATGAAAGTCGGTAACACCCGAAGCCGGTGGCCTAACCCCTTGTGGGAGGGAGCCGTCGAAGGTGGGATCGGCGATTGGGACGAAGTCGTAACAAGGTAGCC

**No.4 *Nocardia farcinia* 1449bp**

CACATGCAAGTCGAGCGGTAAGGCCCTTCGGGGTACACGAGCGGCGAACGGGTGAGTAACACGTGGGTGATCTGCCCTGTACTTCGGGATAAGCCTGGGAAACTGGGTCTAATACCGGATATGACCTTACATCGCATGGTGTTTGGTGGAAAGATTTATCGGTACAGGATGGGCCCGCGGCCTATCAGCTTGTTGGTGGGGTAATGGCCTACCAAGGCGACGACGGGTAGCCGGCCTGAGAGGGCGACCGGCCACACTGGGACTGAGACACGGCCCAGACTCCTACGGGAGGCAGCAGTGGGGAATATTGCACAATGGGCGAAAGCCTGATGCAGCGACGCCGCGTGAGGGATGACGGCCTTCGGGTTGTAAACCTCTTTCGACAGGGACGAAGCGCAAGTGACGGTACCTGTAGAAGAAGCACCGGCCAACTACGTGCCAGCAGCCGCGGTAATACGTAGGGTGCGAGCGTTGTCCGGAATTACTGGGCGTAAAGAGCTTGTAGGCGGTTTGTCGCGTCGTCCGTGAAAACTTGGGGCTCAACCCCAAGCTTGCGGGCGATACGGGCAGACTTGAGTACTGCAGGGGAGACTGGAATTCCTGGTGTAGCGGTGAAATGCGCAGATATCAGGAGGAACACCGGTGGCGAAGGCGGGTCTCTGGGCAGTAACTGACGCTGAGAAGCGAAAGCGTGGGTAGCGAACAGGATTAGATACCCTGGTAGTCCACGCCGTAAACGGTGGGCGCTAGGTGTGGGTTTCCTTCCACGGGATCCGTGCCGTAGCTAACGCATTAAGCGCCCCGCCTGGGGAGTACGGCCGCAAGGCTAAAACTCAAAGGAATTGACGGGGGCCCGCACAAGCGGCGGAGCATGTGGATTAATTCGATGCAACGCGAAGAACCTTACCTGGGTTTGACATACACCGGAAACCTGCAGAGATGTAGGCCCCCTTGTGGTCGGTGTACAGGTGGTGCATGGCTGTCGTCAGCTCGTGTCGTGAGATGTTGGGTTAAGTCCCGCAACGAGCGCAACCCTTGTCCTGTGTTGCCAGCGCGTTATGGCGGGGACTCGCAGGAGACTGCCGGGGTCAACTCGGAGGAAGGTGGGGACGACGTCAAGTCATCATGCCCCTTATGTCCAGGGCTTCACACATGCTACAATGGCCGGTACAGAGGGCTGCGATACCGTGAGGTGGAGCGAATCCCTTAAAGCCGGTCTCAGTTCGGATCGGGGTCTGCAACTCGACCCCGTGAAGTTGGAGTCGCTAGTAATCGCAGATCAGCAACGCTGCGGTGAATACGTTCCCGGGCCTTGTACACACCGCCCGTCACGTCATGAAAGTCGGTAACACCCGAAGCCGGTGGCCTAACCCCTTGTGGGAGGGAGCCGTCGAAGGTGGGATCGGCGATTGGGACGAAGTCGTAACAAGGTAGCCGTACCGGAAGGTG

**No.5 *Nocardia otitidiscaviarum* 1459bp**

CCTGGCTCAGGACGAACGCTGGCGGCGTGCTTAACACATGCAAGTCGAGCGGTAAGGCCCTTCGGGGTACACGAGCGGCGAACGGGTGAGTAACACGTGGGTGATCTGCCTCGCACTCTGGGATAAGCCTGGGAAACTGGGTCTAATACCGGATATGACCACGAATCGCATGGTTTGTGGTGGAAAGGTTTACTGGTGCGAGATGGGCCCGCGGCCTATCAGCTTGTTGGTGGGGTAACGGCCTACCAAGGCGACGACGGGTAGCCGGCCTGAGAGGGCGACCGGCCACACTGGGACTGAGACACGGCCCAGACTCCTACGGGAGGCAGCAGTGGGGAATATTGCACAATGGGCGAAAGCCTGATGCAGCGACGCCGCGTGAGGGATGACGGCCTTCGGGTTGTAAACCTCTTTCGACAGGGACGAAGCGCAAGTGACGGTACCTGTAGAAGAAGCACCGGCCAACTACGTGCCAGCAGCCGCGGTAATACGTAGGGTGCGAGCGTTGTCCGGAATTACTGGGCGTAAAGAGCTTGTAGGCGGTCTGTCGCGTCGATCGTGAAAACGTGCAGCTCAACTGCGCGCCTGCGGTCGATACGGGCAGACTAGAGTACTTCAGGGGAGACTGGAATTCCTGGTGTAGCGGTGAAATGCGCAGATATCAGGAGGAACACCGGTGGCGAAGGCGGGTCTCTGGGAAGTAACTGACGCTGAGAAGCGAAAGCGTGGGTAGCGAACAGGATTAGATACCCTGGTAGTCCACGCCGTAAACGGTGGGTACTAGGTGTGGGTTTCCTTCCACGGGATCCGTGCCGTAGCTAACGCATTAAGTACCCCGCCTGGGGAGTACGGCCGCAAGGCTAAAACTCAAAGGAATTGACGGGGGCCCGCACAAGCGGCGGAGCATGTGGATTAATTCGATGCAACGCGAAGAACCTTACCTGGGTTTGACATACACCGGAAAGCCGTAGAGATACGGCCCCCCTTGTGGTCGGTGTACAGGTGGTGCATGGCTGTCGTCAGCTCGTGTCGTGAGATGTTGGGTTAAGTCCCGCAACGAGCGCAACCCTTATCTTATGTTGCCAGCGCGTAATGGCGGGGACTCGTGAGAGACTGCCGGGGTCAACTCGGAGGAAGGTGGGGACGACGTCAAGTCATCATGCCCCTTATGTCCAGGGCTTCACACATGCTACAATGGCCGGTACAGAGGGCTGCGATACCGCGAGGTGGAGCGAATCCCTTAAAGCCGGTCTCAGTTCGGATCGGGGTCTGCAACTCGACCCCGTGAAGTTGGAGTCGCTAGTAATCGCAGATCAGCAACGCTGCGGTGAATACGTTCCCGGGCCTTGTACACACCGCCCGTCACGTCATGAAAGTCGGTAACACCCGAAGCCGGTGGCCTAACCCTCTGGGAGGGAGCCGTCGAAGGTGGGATTGGCGATTGGGACGAAGTCGTAAC

**No.6 *Nocardia cyriacigeorgica* 1489bp**

CTGGCTCAGGACGAACGCTGGCGGCGTGCTTAACACATGCAAGTCGAGCGGTAAGGCCCTTCGGGGTACACGAGCGGCGAACGGGTGAGTAACACGTGGGTGATCTGCCTCGCACTCTGGGATAAGCCTGGGAAACTGGGTCTAATACCGGATATGACCTTACATCGCATGGTGTTTGGTGGAAAGATTTATCGGTGCGAGATGGGCCCGCGGCCTATCAGCTTGTTGGTGGGGTAATGGCCTACCAAGGCGACGACGGGTAGCCGGCCTGAGAGGGCGACCGGCCACACTGGGACTGAGACACGGCCCAGACTCCTACGGGAGGCAGCAGTGGGGAATATTGCACAATGGGCGAAAGCCTGATGCAGCGACGCCGCGTGAGGGATGACGGCCTTCGGGTTGTAAACCTCTTTCGACAGGGACGAAGCGCAAGTGACGGTACCTGTAGAAGAAGCACCGGCCAACTACGTGCCAGCAGCCGCGGTAATACGTAGGGTGCGAGCGTTGTCCGGAATTACTGGGCGTAAAGAGCTTGTAGGCGGCTTGTCGCGTCGATCGTGAAAACTTGGGGCTCAACCCCAAGCTTGCGGTCGATACGGGCAGGCTTGAGTACTTCAGGGGAGACTGGAATTCCTGGTGTAGCGGTGAAATGCGCAGATATCAGGAGGAACACCGGTGGCGAAGGCGGGTCTCTGGGAAGTAACTGACGCTGAGAAGCGAAAGCGTGGGTAGCGAACAGGATTAGATACCCTGGTAGTCCACGCCGTAAACGGTGGGTACTAGGTGTGGGTTTCCTTCCACGGGATCCGTGCCGTAGCTAACGCATTAAGTACCCCGCCTGGGGAGTACGGCCGCAAGGCTAAAACTCAAAGGAATTGACGGGGGCCCGCACAAGCGGCGGAGCATGTGGATTAATTCGATGCAACGCGAAGAACCTTACCTGGGTTTGACATACACCGGAAACCTGCAGAGATGTAGGCCCCCTTGTGGTCGGTGTACAGGTGGTGCATGGCTGTCGTCAGCTCGTGTCGTGAGATGTTGGGTTAAGTCCCGCAACGAGCGCAACCCTTATCTTATGTTGCCAGCGCGTAATGGCGGGGACTCGTGAGAGACTGCCGGGGTCAACTCGGAGGAAGGTGGGGACGACGTCAAGTCATCATGCCCCTTATGTCCAGGGCTTCACACATGCTACAATGGCCGGTACAGAGGGCTGCGATACCGTGAGGTGGAGCGAATCCCTTAAAGCCGGTCTCAGTTCGGATCGGGGTCTGCAACTCGACCCCGTGAAGTTGGAGTCGCTAGTAATCGCAGATCAGCAACGCTGCGGTGAATACGTTCCCGGGCCTTGTACACACCGCCCGTCACGTCATGAAAGTCGGTAACACCCGAAGCCGGTGGCCTAACCCCTTGTGGGAGGGAGCCGTCGAAGGTGGGATCGGCGATTGGGACGAAGTCGTAACAAGGTAGCCGTACCGGAAGGTGCGGCTGGAAGGT

**No.7 *Nocardia brasiliensis***  **1463bp**

CTGGCTCAGGACGAACGCTGGCGGCGTGCTTAACACATGCAAGTCGAGCGGTAAGGCCCTTCGGGGTACACGAGCGGCGAACGGGTGAGTAACACGTGGGTGATCTGCCTCGCACTTCGGGATAAGCCTGGGAAACTGGGTCTAATACCGGATATGACCTTTCAGTGCATGCTGTTGGGTGGAAAGATTTATCGGTGCGAGATGGGCCCGCGGCCTATCAGCTTGTTGGCGGGGTAACGGCCCACCAAGGCGACGACGGGTAGCCGACCTGAGAGGGTGACCGGCCACACTGGGACTGAGACACGGCCCAGACTCCTACGGGAGGCAGCAGTGGGGAATATTGCACAATGGGCGAAAGCCTGATGCAGCGACGCCGCGTGAGGGATGACGGCCTTCGGGTTGTAAACCTCTTTCGACAGGGACGAAGCGCAAGTGACGGTACCTGTAGAAGAAGCACCGGCCAACTACGTGCCAGCAGCCGCGGTAATACGTAGGGTGCGAGCGTTGTCCGGAATTACTGGGCGTAAAGAGCTTGTAGGCGGTTTGTCGCGTCGTCCGTGAAAACTTGGGGCTCAACCCCAAGCTTGCGGGCGATACGGGCAGACTTGAGTACTTCAGGGGAGACTGGAATTCCTGGTGTAGCGGTGAAATGCGCAGATATCAGGAGGAACACCGGTGGCGAAGGCGGGTCTCTGGGAAGTAACTGACGCTGAGAAGCGAAAGCGTGGGTAGCGAACAGGATTAGATACCCTGGTAGTCCACGCCGTAAACGGTGGGTACTAGGTGTGGGTTTCCTTCCACGGGATCCGTGCCGTAGCTAACGCATTAAGTACCCCGCCTGGGGAGTACGGCCGCAAGGCTAAAACTCAAAGGAATTGACGGGGGCCCGCACAAGCGGCGGAGCATGTGGATTAATTCGATGCAACGCGAAGAACCTTACCTGGGTTTGACATACACCGGAAACCTGCAGAGATGTAGGCCCCCTTGTGGTCGGTGTACAGGTGGTGCATGGCTGTCGTCAGCTCGTGTCGTGAGATGTTGGGTTAAGTCCCGCAACGAGCGCAACCCTTGTCCTGTGTTGCCAGCGGATTATGCCGGGGACTCGCAGGAGACTGCCGGGGTCAACTCGGAGGAAGGTGGGGACGACGTCAAGTCATCATGCCCCTTATGTCCAGGGCTTCACACATGCTACAATGGCCGGTACAGAGGGCTGCGATACCGTGAGGTGGAGCGAATCCCTTAAAGCCGGTCTCAGTTCGGATCGGGGTCTGCAACTCGACCCCGTGAAGTTGGAGTCGCTAGTAATCGCAGATCAGCAACGCTGCGGTGAATACGTTCCCGGGCCTTGTACACACCGCCCGTCACGTCATGAAAGTCGGTAACACCCGAAGCCGGTGGCCTAACCCCTTGTGGGAGGGAGCCGTCGAAGGTGGGATTGGCGATTGGGACGAAGTCGTAACAAG

**No.8 *Nocardia cyriacigeorgica* 1496bp**

CAGGACGCTGGCTCAGGACGAACGCTGGCGGCGTGCTTAACACATGCAAGTCGAGCGGTAAGGCCCTTCGGGGTACACGAGCGGCGAACGGGTGAGTAACACGTGGGTGATCTGCCTCGCACTCTGGGATAAGCCTGGGAAACTGGGTCTAATACCGGATATGACCTTACATCGCATGGTGTTTGGTGGAAAGATTTATCGGTGCGAGATGGGCCCGCGGCCTATCAGCTTGTTGGTGGGGTAATGGCCTACCAAGGCGACGACGGGTAGCCGGCCTGAGAGGGCGACCGGCCACACTGGGACTGAGACACGGCCCAGACTCCTACGGGAGGCAGCAGTGGGGAATATTGCACAATGGGCGAAAGCCTGATGCAGCGACGCCGCGTGAGGGATGACGGCCTTCGGGTTGTAAACCTCTTTCGACAGGGACGAAGCGCAAGTGACGGTACCTGTAGAAGAAGCACCGGCCAACTACGTGCCAGCAGCCGCGGTAATACGTAGGGTGCGAGCGTTGTCCGGAATTACTGGGCGTAAAGAGCTTGTAGGCGGCTTGTCGCGTCGATCGTGAAAACTTGGGGCTCAACCCCAAGCTTGCGGTCGATACGGGCAGGCTTGAGTACTTCAGGGGAGACTGGAATTCCTGGTGTAGCGGTGAAATGCGCAGATATCAGGAGGAACACCGGTGGCGAAGGCGGGTCTCTGGGAAGTAACTGACGCTGAGAAGCGAAAGCGTGGGTAGCGAACAGGATTAGATACCCTGGTAGTCCACGCCGTAAACGGTGGGTACTAGGTGTGGGTTTCCTTCCACGGGATCCGTGCCGTAGCTAACGCATTAAGTACCCCGCCTGGGGAGTACGGCCGCAAGGCTAAAACTCAAAGGAATTGACGGGGGCCCGCACAAGCGGCGGAGCATGTGGATTAATTCGATGCAACGCGAAGAACCTTACCTGGGTTTGACATACACCGGAAACCTGCAGAGATGTAGGCCCCCTTGTGGTCGGTGTACAGGTGGTGCATGGCTGTCGTCAGCTCGTGTCGTGAGATGTTGGGTTAAGTCCCGCAACGAGCGCAACCCTTATCTTATGTTGCCAGCGCGTAATGGCGGGGACTCGTGAGAGACTGCCGGGGTCAACTCGGAGGAAGGTGGGGACGACGTCAAGTCATCATGCCCCTTATGTCCAGGGCTTCACACATGCTACAATGGCCGGTACAGAGGGCTGCGATACCGTGAGGTGGAGCGAATCCCTTAAAGCCGGTCTCAGTTCGGATCGGGGTCTGCAACTCGACCCCGTGAAGTTGGAGTCGCTAGTAATCGCAGATCAGCAACGCTGCGGTGAATACGTTCCCGGGCCTTGTACACACCGCCCGTCACGTCATGAAAGTCGGTAACACCCGAAGCCGGTGGCCTAACCCCTTGTGGGAGGGAGCCGTCGAAGGTGGGATCGGCGATTGGGACGAAGTCGTAACAAGGTAGCCGTACCGGAAGGTGCGGCTGG

**No.9 *Nocardia cyriacigeorgica* 1441bp**

CTCAGGACGAACGCTGGCGGCGTGCTTAACACATGCAAGTCGAGCGGTAAGGCCCTTCGGGGTACACGAGCGGCGAACGGGTGAGTAACACGTGGGTGATCTGCCTCGCACTCTGGGATAAGCCTGGGAAACTGGGTCTAATACCGGATATGACCTTACATCGCATGGTGTTTGGTGGAAAGATTTATCGGTGCGAGATGGGCCCGCGGCCTATCAGCTTGTTGGTGGGGTAATGGCCTACCAAGGCGACGACGGGTAGCCGGCCTGAGAGGGCGACCGGCCACACTGGGACTGAGACACGGCCCAGACTCCTACGGGAGGCAGCAGTGGGGAATATTGCACAATGGGCGAAAGCCTGATGCAGCGACGCCGCGTGAGGGATGACGGCCTTCGGGTTGTAAACCTCTTTCGACAGGGACGAAGCGCAAGTGACGGTACCTGTAGAAGAAGCACCGGCCAACTACGTGCCAGCAGCCGCGGTAATACGTAGGGTGCGAGCGTTGTCCGGAATTACTGGGCGTAAAGAGCTTGTAGGCGGCTTGTCGCGTCGATCGTGAAAACTTGGGGCTCAACCCCAAGCTTGCGGTCGATACGGGCAGGCTTGAGTACTTCAGGGGAGACTGGAATTCCTGGTGTAGCGGTGAAATGCGCAGATATCAGGAGGAACACCGGTGGCGAAGGCGGGTCTCTGGGAAGTAACTGACGCTGAGAAGCGAAAGCGTGGGTAGCGAACAGGATTAGATACCCTGGTAGTCCACGCCGTAAACGGTGGGTACTAGGTGTGGGTTTCCTTCCACGGGATCCGTGCCGTAGCTAACGCATTAAGTACCCCGCCTGGGGAGTACGGCCGCAAGGCTAAAACTCAAAGGAATTGACGGGGGCCCGCACAAGCGGCGGAGCATGTGGATTAATTCGATGCAACGCGAAGAACCTTACCTGGGTTTGACATACACCGGAAACCTGCAGAGATGTAGGCCCCCTTGTGGTCGGTGTACAGGTGGTGCATGGCTGTCGTCAGCTCGTGTCGTGAGATGTTGGGTTAAGTCCCGCAACGAGCGCAACCCTTATCTTATGTTGCCAGCGCGTAATGGCGGGGACTCGTGAGAGACTGCCGGGGTCAACTCGGAGGAAGGTGGGGACGACGTCAAGTCATCATGCCCCTTATGTCCAGGGCTTCACACATGCTACAATGGCCGGTACAGAGGGCTGCGATACCGTGAGGTGGAGCGAATCCCTTAAAGCCGGTCTCAGTTCGGATCGGGGTCTGCAACTCGACCCCGTGAAGTTGGAGTCGCTAGTAATCGCAGATCAGCAACGCTGCGGTGAATACGTTCCCGGGCCTTGTACACACCGCCCGTCACGTCATGAAAGTCGGTAACACCCGAAGCCGGTGGCCTAACCCCTTGTGGGAGGGAGCCGTCGAAGGTGGGATCGGCGATTG

**No.10 *Nocardia cyriacigeorgica* 1452bp**

GGACGAACGCTGGCGGCGTGCTTAACACATGCAAGTCGAGCGGTAAGGCCCTTCGGGGTACACGAGCGGCGAACGGGTGAGTAACACGTGGGTGATCTGCCTCGCACTCTGGGATAAGCCTGGGAAACTGGGTCTAATACCGGATATGACCTTACATCGCATGGTGTTTGGTGGAAAGATTTATCGGTGCGAGATGGGCCCGCGGCCTATCAGCTTGTTGGTGGGGTAATGGCCTACCAAGGCGACGACGGGTAGCCGGCCTGAGAGGGCGACCGGCCACACTGGGACTGAGACACGGCCCAGACTCCTACGGGAGGCAGCAGTGGGGAATATTGCACAATGGGCGAAAGCCTGATGCAGCGACGCCGCGTGAGGGATGACGGCCTTCGGGTTGTAAACCTCTTTCGACAGGGACGAAGCGCAAGTGACGGTACCTGTAGAAGAAGCACCGGCCAACTACGTGCCAGCAGCCGCGGTAATACGTAGGGTGCGAGCGTTGTCCGGAATTACTGGGCGTAAAGAGCTTGTAGGCGGCTTGTCGCGTCGATCGTGAAAACTTGGGGCTCAACCCCAAGCTTGCGGTCGATACGGGCAGGCTTGAGTACTTCAGGGGAGACTGGAATTCCTGGTGTAGCGGTGAAATGCGCAGATATCAGGAGGAACACCGGTGGCGAAGGCGGGTCTCTGGGAAGTAACTGACGCTGAGAAGCGAAAGCGTGGGTAGCGAACAGGATTAGATACCCTGGTAGTCCACGCCGTAAACGGTGGGTACTAGGTGTGGGTTTCCTTCCACGGGATCCGTGCCGTAGCTAACGCATTAAGTACCCCGCCTGGGGAGTACGGCCGCAAGGCTAAAACTCAAAGGAATTGACGGGGGCCCGCACAAGCGGCGGAGCATGTGGATTAATTCGATGCAACGCGAAGAACCTTACCTGGGTTTGACATACACCGGAAACCTGCAGAGATGTAGGCCCCCTTGTGGTCGGTGTACAGGTGGTGCATGGCTGTCGTCAGCTCGTGTCGTGAGATGTTGGGTTAAGTCCCGCAACGAGCGCAACCCTTATCTTATGTTGCCAGCGCGTAATGGCGGGGACTCGTGAGAGACTGCCGGGGTCAACTCGGAGGAAGGTGGGGACGACGTCAAGTCATCATGCCCCTTATGTCCAGGGCTTCACACATGCTACAATGGCCGGTACAGAGGGCTGCGATACCGTGAGGTGGAGCGAATCCCTTAAAGCCGGTCTCAGTTCGGATCGGGGTCTGCAACTCGACCCCGTGAAGTTGGAGTCGCTAGTAATCGCAGATCAGCAACGCTGCGGTGAATACGTTCCCGGGCCTTGTACACACCGCCCGTCACGTCATGAAAGTCGGTAACACCCGAAGCCGGTGGCCTAACCCCTTGTGGGAGGGAGCCGTCGAAGGTGGGATCGGCGATTGGGACGAAGTCGTAAC

**No.11 *Nocardia cyriacigeorgica* 1481bp**

GGACGAACGCTGGCGGCGTGCTTAACACATGCAAGTCGAGCGGTAAGGCCCTTCGGGGTACACGAGCGGCGAACGGGTGAGTAACACGTGGGTGATCTGCCTCGCACTCTGGGATAAGCCTGGGAAACTGGGTCTAATACCGGATATGACCTTACATCGCATGGTGTTTGGTGGAAAGATTTATCGGTGCGAGATGGGCCCGCGGCCTATCAGCTTGTTGGTGGGGTAATGGCCTACCAAGGCGACGACGGGTAGCCGGCCTGAGAGGGCGACCGGCCACACTGGGACTGAGACACGGCCCAGACTCCTACGGGAGGCAGCAGTGGGGAATATTGCACAATGGGCGAAAGCCTGATGCAGCGACGCCGCGTGAGGGATGACGGCCTTCGGGTTGTAAACCTCTTTCGACAGGGACGAAGCGCAAGTGACGGTACCTGTAGAAGAAGCACCGGCCAACTACGTGCCAGCAGCCGCGGTAATACGTAGGGTGCGAGCGTTGTCCGGAATTACTGGGCGTAAAGAGCTTGTAGGCGGCTTGTCGCGTCGATCGTGAAAACTTGGGGCTCAACCCCAAGCTTGCGGTCGATACGGGCAGGCTTGAGTACTTCAGGGGAGACTGGAATTCCTGGTGTAGCGGTGAAATGCGCAGATATCAGGAGGAACACCGGTGGCGAAGGCGGGTCTCTGGGAAGTAACTGACGCTGAGAAGCGAAAGCGTGGGTAGCGAACAGGATTAGATACCCTGGTAGTCCACGCCGTAAACGGTGGGTACTAGGTGTGGGTTTCCTTCCACGGGATCCGTGCCGTAGCTAACGCATTAAGTACCCCGCCTGGGGAGTACGGCCGCAAGGCTAAAACTCAAAGGAATTGACGGGGGCCCGCACAAGCGGCGGAGCATGTGGATTAATTCGATGCAACGCGAAGAACCTTACCTGGGTTTGACATACACCGGAAACCTGCAGAGATGTAGGCCCCCTTGTGGTCGGTGTACAGGTGGTGCATGGCTGTCGTCAGCTCGTGTCGTGAGATGTTGGGTTAAGTCCCGCAACGAGCGCAACCCTTATCTTATGTTGCCAGCGCGTAATGGCGGGGACTCGTGAGAGACTGCCGGGGTCAACTCGGAGGAAGGTGGGGACGACGTCAAGTCATCATGCCCCTTATGTCCAGGGCTTCACACATGCTACAATGGCCGGTACAGAGGGCTGCGATACCGTGAGGTGGAGCGAATCCCTTAAAGCCGGTCTCAGTTCGGATCGGGGTCTGCAACTCGACCCCGTGAAGTTGGAGTCGCTAGTAATCGCAGATCAGCAACGCTGCGGTGAATACGTTCCCGGGCCTTGTACACACCGCCCGTCACGTCATGAAAGTCGGTAACACCCGAAGCCGGTGGCCTAACCCCTTGTGGGAGGGAGCCGTCGAAGGTGGGATCGGCGATTGGGACGAAGTCGTAACAAGGTAGCCGTACCGGAAGGTGCGGCTGG

**No.12 *Nocardia farcinia* 1483bp**

CTGGCGGCGTGCTTAACACATGCAAGTCGAGCGGTAAGGCCCTTCGGGGTACACGAGCGGCGAACGGGTGAGTAACACGTGGGTGATCTGCCCTGTACTTCGGGATAAGCCTGGGAAACTGGGTCTAATACCGGATATGACCTTACATCGCATGGTGTTTGGTGGAAAGATTTATCGGTACAGGATGGGCCCGCGGCCTATCAGCTTGTTGGTGGGGTAATGGCCTACCAAGGCGACGACGGGTAGCCGGCCTGAGAGGGCGACCGGCCACACTGGGACTGAGACACGGCCCAGACTCCTACGGGAGGCAGCAGTGGGGAATATTGCACAATGGGCGAAAGCCTGATGCAGCGACGCCGCGTGAGGGATGACGGCCTTCGGGTTGTAAACCTCTTTCGACAGGGACGAAGCGCAAGTGACGGTACCTGTAGAAGAAGCACCGGCCAACTACGTGCCAGCAGCCGCGGTAATACGTAGGGTGCGAGCGTTGTCCGGAATTACTGGGCGTAAAGAGCTTGTAGGCGGTTTGTCGCGTCGTCCGTGAAAACTTGGGGCTCAACCCCAAGCTTGCGGGCGATACGGGCAGACTTGAGTACTGCAGGGGAGACTGGAATTCCTGGTGTAGCGGTGAAATGCGCAGATATCAGGAGGAACACCGGTGGCGAAGGCGGGTCTCTGGGCAGTAACTGACGCTGAGAAGCGAAAGCGTGGGTAGCGAACAGGATTAGATACCCTGGTAGTCCACGCCGTAAACGGTGGGCGCTAGGTGTGGGTTTCCTTCCACGGGATCCGTGCCGTAGCTAACGCATTAAGCGCCCCGCCTGGGGAGTACGGCCGCAAGGCTAAAACTCAAAGGAATTGACGGGGGCCCGCACAAGCGGCGGAGCATGTGGATTAATTCGATGCAACGCGAAGAACCTTACCTGGGTTTGACATACACCGGAAACCTGCAGAGATGTAGGCCCCCTTGTGGTCGGTGTACAGGTGGTGCATGGCTGTCGTCAGCTCGTGTCGTGAGATGTTGGGTTAAGTCCCGCAACGAGCGCAACCCTTGTCCTGTGTTGCCAGCGCGTTATGGCGGGGACTCGCAGGAGACTGCCGGGGTCAACTCGGAGGAAGGTGGGGACGACGTCAAGTCATCATGCCCCTTATGTCCAGGGCTTCACACATGCTACAATGGCCGGTACAGAGGGCTGCGATACCGTGAGGTGGAGCGAATCCCTTAAAGCCGGTCTCAGTTCGGATCGGGGTCTGCAACTCGACCCCGTGAAGTTGGAGTCGCTAGTAATCGCAGATCAGCAACGCTGCGGTGAATACGTTCCCGGGCCTTGTACACACCGCCCGTCACGTCATGAAAGTCGGTAACACCCGAAGCCGGTGGCCTAACCCCTTGTGGGAGGGAGCCGTCGAAGGTGGGATCGGCGATTGGGACGAAGTCGTAACAAGGTAGCC

**No.13 *Nocardia cyriacigeorgica* 1471bp**

TGGCGGCGTGCTTAACACATGCAAGTCGAGCGGTAAGGCCCTTCGGGGTACACGAGCGGCGAACGGGTGAGTAACACGTGGGTGATCTGCCTCGCACTCTGGGATAAGCCTGGGAAACTGGGTCTAATACCGGATATGACCTTACATCGCATGGTGTTTGGTGGAAAGATTTATCGGTGCGAGATGGGCCCGCGGCCTATCAGCTTGTTGGTGGGGTAATGGCCTACCAAGGCGACGACGGGTAGCCGGCCTGAGAGGGCGACCGGCCACACTGGGACTGAGACACGGCCCAGACTCCTACGGGAGGCAGCAGTGGGGAATATTGCACAATGGGCGAAAGCCTGATGCAGCGACGCCGCGTGAGGGATGACGGCCTTCGGGTTGTAAACCTCTTTCGACAGGGACGAAGCGCAAGTGACGGTACCTGTAGAAGAAGCACCGGCCAACTACGTGCCAGCAGCCGCGGTAATACGTAGGGTGCGAGCGTTGTCCGGAATTACTGGGCGTAAAGAGCTTGTAGGCGGCTTGTCGCGTCGATCGTGAAAACTTGGGGCTCAACCCCAAGCTTGCGGTCGATACGGGCAGGCTTGAGTACTTCAGGGGAGACTGGAATTCCTGGTGTAGCGGTGAAATGCGCAGATATCAGGAGGAACACCGGTGGCGAAGGCGGGTCTCTGGGAAGTAACTGACGCTGAGAAGCGAAAGCGTGGGTAGCGAACAGGATTAGATACCCTGGTAGTCCACGCCGTAAACGGTGGGTACTAGGTGTGGGTTTCCTTCCACGGGATCCGTGCCGTAGCTAACGCATTAAGTACCCCGCCTGGGGAGTACGGCCGCAAGGCTAAAACTCAAAGGAATTGACGGGGGCCCGCACAAGCGGCGGAGCATGTGGATTAATTCGATGCAACGCGAAGAACCTTACCTGGGTTTGACATACACCGGAAACCTGCAGAGATGTAGGCCCCCTTGTGGTCGGTGTACAGGTGGTGCATGGCTGTCGTCAGCTCGTGTCGTGAGATGTTGGGTTAAGTCCCGCAACGAGCGCAACCCTTATCTTATGTTGCCAGCGCGTAATGGCGGGGACTCGTGAGAGACTGCCGGGGTCAACTCGGAGGAAGGTGGGGACGACGTCAAGTCATCATGCCCCTTATGTCCAGGGCTTCACACATGCTACAATGGCCGGTACAGAGGGCTGCGATACCGTGAGGTGGAGCGAATCCCTTAAAGCCGGTCTCAGTTCGGATCGGGGTCTGCAACTCGACCCCGTGAAGTTGGAGTCGCTAGTAATCGCAGATCAGCAACGCTGCGGTGAATACGTTCCCGGGCCTTGTACACACCGCCCGTCACGTCATGAAAGTCGGTAACACCCGAAGCCGGTGGCCTAACCCCTTGTGGGAGGGAGCCGTCGAAGGTGGGATCGGCGATTGGGACGAAGTCGTAACAAGGTAGCCGTACCGGAAGGTGCGGCTGG

**No.14 *Nocardia farcinia* 1454bp**

TTAACACATGCAAGTCGAGCGGTAAGGCCCTTCGGGGTACACGAGCGGCGAACGGGTGAGTAACACGTGGGTGATCTGCCCTGTACTTCGGGATAAGCCTGGGAAACTGGGTCTAATACCGGATATGACCTTACATCGCATGGTGTTTGGTGGAAAGATTTATCGGTACAGGATGGGCCCGCGGCCTATCAGCTTGTTGGTGGGGTAATGGCCTACCAAGGCGACGACGGGTAGCCGGCCTGAGAGGGCGACCGGCCACACTGGGACTGAGACACGGCCCAGACTCCTACGGGAGGCAGCAGTGGGGAATATTGCACAATGGGCGAAAGCCTGATGCAGCGACGCCGCGTGAGGGATGACGGCCTTCGGGTTGTAAACCTCTTTCGACAGGGACGAAGCGCAAGTGACGGTACCTGTAGAAGAAGCACCGGCCAACTACGTGCCAGCAGCCGCGGTAATACGTAGGGTGCGAGCGTTGTCCGGAATTACTGGGCGTAAAGAGCTTGTAGGCGGTTTGTCGCGTCGTCCGTGAAAACTTGGGGCTCAACCCCAAGCTTGCGGGCGATACGGGCAGACTTGAGTACTGCAGGGGAGACTGGAATTCCTGGTGTAGCGGTGAAATGCGCAGATATCAGGAGGAACACCGGTGGCGAAGGCGGGTCTCTGGGCAGTAACTGACGCTGAGAAGCGAAAGCGTGGGTAGCGAACAGGATTAGATACCCTGGTAGTCCACGCCGTAAACGGTGGGCGCTAGGTGTGGGTTTCCTTCCACGGGATCCGTGCCGTAGCTAACGCATTAAGCGCCCCGCCTGGGGAGTACGGCCGCAAGGCTAAAACTCAAAGGAATTGACGGGGGCCCGCACAAGCGGCGGAGCATGTGGATTAATTCGATGCAACGCGAAGAACCTTACCTGGGTTTGACATACACCGGAAACCTGCAGAGATGTAGGCCCCCTTGTGGTCGGTGTACAGGTGGTGCATGGCTGTCGTCAGCTCGTGTCGTGAGATGTTGGGTTAAGTCCCGCAACGAGCGCAACCCTTGTCCTGTGTTGCCAGCGCGTTATGGCGGGGACTCGCAGGAGACTGCCGGGGTCAACTCGGAGGAAGGTGGGGACGACGTCAAGTCATCATGCCCCTTATGTCCAGGGCTTCACACATGCTACAATGGCCGGTACAGAGGGCTGCGATACCGTGAGGTGGAGCGAATCCCTTAAAGCCGGTCTCAGTTCGGATCGGGGTCTGCAACTCGACCCCGTGAAGTTGGAGTCGCTAGTAATCGCAGATCAGCAACGCTGCGGTGAATACGTTCCCGGGCCTTGTACACACCGCCCGTCACGTCATGAAAGTCGGTAACACCCGAAGCCGGTGGCCTAACCCCTTGTGGGAGGGAGCCGTCGAAGGTGGGATCGGCGATTGGGACGAAGTCGTAACAAGGTAGCCGTACCGGAAGGTGC
